# Mertensene, a Halogenated Monoterpene, Induces G2/M Cell Cycle Arrest and Caspase Dependent Apoptosis of Human Colon Adenocarcinoma HT29 Cell Line through the Modulation of ERK-1/-2, AKT and NF-κB Signaling

**DOI:** 10.3390/md15070221

**Published:** 2017-07-20

**Authors:** Safa Tarhouni-Jabberi, Ons Zakraoui, Efstathia Ioannou, Ichrak Riahi-Chebbi, Meriam Haoues, Vassilios Roussis, Riadh Kharrat, Khadija Essafi-Benkhadir

**Affiliations:** 1 Institut Pasteur de Tunis, Laboratoire de Toxines Alimentaires, LR11IPT08 Laboratoire des Venins et Molécules Thérapeutiques, 1002 Tunis, Tunisia; satarhouni@yahoo.fr; 2 Faculté des Sciences de Bizerte, Université de Carthage, 1002 Tunis, Tunisia; 3 Institut Pasteur de Tunis, LR11IPT04 Laboratoire d’Epidémiologie Moléculaire et de Pathologie Expérimentale Appliquée Aux Maladies Infectieuses, 1002 Tunis, Tunisia; zakraoui-ons@hotmail.fr (O.Z.); ichrakriahi@live.com (I.R.-C.); 4 Université de Tunis El Manar, 1068 Tunis, Tunisia; 5 Department of Pharmacognosy and Chemistry of Natural Products, Faculty of Pharmacy, National and Kapodistrian University of Athens, Panepistimiopolis Zografou, Athens 15771, Greece; eioannou@pharm.uoa.gr (E.I.); roussis@pharm.uoa.gr (V.R.); 6 Institut Pasteur de Tunis, LR11IPT02 Laboratoire de Recherche sur la Transmission, le Contrôle et l’Immunobiologie des Infections, 1002 Tunis, Tunisia; hwesmeriam@hotmail.com

**Keywords:** *Pterocladiella capillacea*, mertensene, colon cancer, cell cycle arrest, apoptosis, cellular effectors

## Abstract

Conventional treatment of advanced colorectal cancer is associated with tumor resistance and toxicity towards normal tissues. Therefore, development of effective anticancer therapeutic alternatives is still urgently required. Nowadays, marine secondary metabolites have been extensively investigated due to the fact that they frequently exhibit anti-tumor properties. However, little attention has been given to terpenoids isolated from seaweeds. In this study, we isolated the halogenated monoterpene mertensene from the red alga *Pterocladiella capillacea* (S.G. Gmelin) Santelices and Hommersand and we highlight its inhibitory effect on the viability of two human colorectal adenocarcinoma cell lines HT29 and LS174. Interestingly, exposure of HT29 cells to different concentrations of mertensene correlated with the activation of MAPK ERK-1/-2, Akt and NF-κB pathways. Moreover, mertensene-induced G2/M cell cycle arrest was associated with a decrease in the phosphorylated forms of the anti-tumor transcription factor *p53*, retinoblastoma protein (Rb), cdc2 and chkp2. Indeed, a reduction of the cellular level of cyclin-dependent kinases CDK2 and CDK4 was observed in mertensene-treated cells. We also demonstrated that mertensene triggers a caspase-dependent apoptosis in HT29 cancer cells characterized by the activation of caspase-3 and the cleavage of poly (ADP-ribose) polymerase (PARP). Besides, the level of death receptor-associated protein TRADD increased significantly in a concentration-dependent manner. Taken together, these results demonstrate the potential of mertensene as a drug candidate for the treatment of colon cancer.

## 1. Introduction

Cancer is a hyper-proliferative disorder that involves transformation, dysregulation of apoptosis, proliferation, invasion, angiogenesis, and metastasis and it is classified among the primary causes of mortality worldwide [1]. The colorectal cancer ranks as the most frequently diagnosed type of cancer with an increasing annual incidence and mortality rate [2]. Therefore, improving the efficiency of the currently available therapies and reducing their adverse effects during treatment through development of new therapeutic alternatives remains compulsory.

Natural products represent a diverse chemical library of bioactive molecules that exhibit anti-tumoral activity [3]. The oceans, covering more than 70% of the earth’s surface and amounting approximately to 95% of the global biosphere, represent an extraordinarily rich source of chemical and biological diversity [4]. Marine chemodiversity, characterized by a wide variety of often unique structures, resulting from different biological processes, such as metabolic pathways, reproductive systems and evolutionary traits, includes compounds of outstanding potency [5]. Metabolites of marine organisms display unique chemical skeletons and interesting functionalities [4,6,7].

Up to date, the majority of the bioactive algal secondary metabolites that comprise a large number of chemical entities have been extracted mainly from red and brown algae and fewer from green algae [8,9]. A number of these metabolites, including terpenoids, acetogenins and phenolic compounds, have exhibited antioxidant, antibacterial, anti-inflammatory, analgesic, antihypertensive and anticancer properties [10]. Besides the pharmacological interest that algal metabolites exhibit, several algal species are known to produce a number of natural products that exhibit important ecological roles as defense and/or signal compounds, while also holding a potential for a wide array of biotechnological applications [11].

According to Scopus database, more than 100 reviews on “alga” and “tumor” have been published to date. Nonetheless, up to now none of the assayed algal compounds has been singled out as a potential anti-cancer agent, with the exception of halomon. Halomon (6(*R*)-bromo-3(*S*)-(bromomethyl)-7-methyl-2,3,7-trichloro-1-octene), a pentahalogenated monoterpene isolated for the first time from the red alga *Portieria hornemannii*, is one of the most well-known algal metabolites that exhibits strong anticancer activity [12]. Unfortunately, the preclinical drug development of halomon has been abandoned due to its failure to show in vivo effects and the lack of a reliable supply source.

A number of cytotoxic analogues of halomon and cyclic and linear unrelated halogenated monoterpenes were later isolated from species of the genus *Plocamium* (*Plocamium cartilagineum* [13], *Plocamium suhrii*, *Plocamium cornutum* [14], and *Plocamium corallorhiza* [15,16]) and characterized for their anti-tumor activity [12,17]. However, further studies are needed to uncover their mechanism of action.

In the present study, efforts have focused on highlighting the antiproliferative effect of mertensene ((1*R*, 2*S*, 4*R*, 5*R*)-2-bromo-4,5-dichloro-1-((*E*)-2-chlorovinyl)-1,5-dimethylcyclohexane), a polyhalogenated monoterpene isolated from the red alga *Pterocladiella capillacea* (S.G. Gmelin) Santelices & Hommersand. This metabolite was first isolated from an unclassified species of *Plocamium* found off the Western Australia coasts [18]. The phytochemical investigations reported on *P. capillacea* are limited and concern the extraction of galactans [19,20,21,22], lectins [23], bromophenols [24], and some methyl ammonium small molecules [25]. To the best of our knowledge, this is the first report describing the isolation of mertensene from *P. capillacea* and the evaluation of its anti-tumor effect against human colorectal adenocarcinoma cell lines. The biochemical and molecular investigations demonstrated that mertensene inhibits the viability of two human colorectal cell lines HT29 and LS174. This antiproliferative effect occurs through cell cycle blockade and the induction of cell apoptosis accompanied with modulation of the MAPK ERK-1/-2, AKT and NF-κB signaling pathways.

## 2. Results

### 2.1. Isolation and Identification of Mertensene

**Figure 1:**
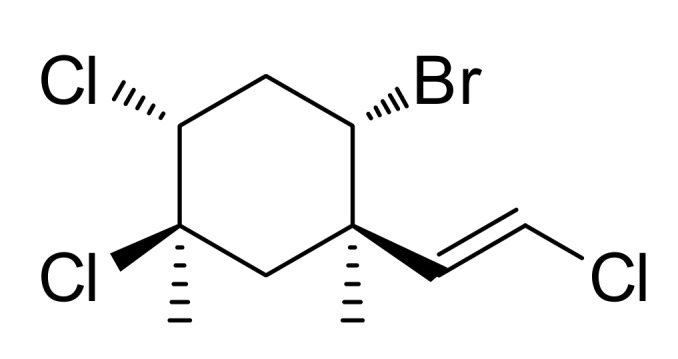
Structure of mertensene.

Mertensene (0.005% dry wt, Figure 1) was purified through a series of chromatographic separations of the red algal extract of *P. capillacea* and its structure was confirmed based on analysis of its NMR and MS spectroscopic data [26]. The absolute configuration of mertensene has been established through single-crystal X-ray crystallographic analysis [18].

The skeleton of mertensene was, for a long time, associated with Plocamium species (order Plocamiales). Thus, our results highlight the presence of mertensene backbone in *P. capillacea* (order Gelidiales).

### 2.2. Mertensene Affects the Viability of HT29 and LS174 Human Colon Adenocarcinoma Cells Independently of Their p53 Status

**Figure 2:**
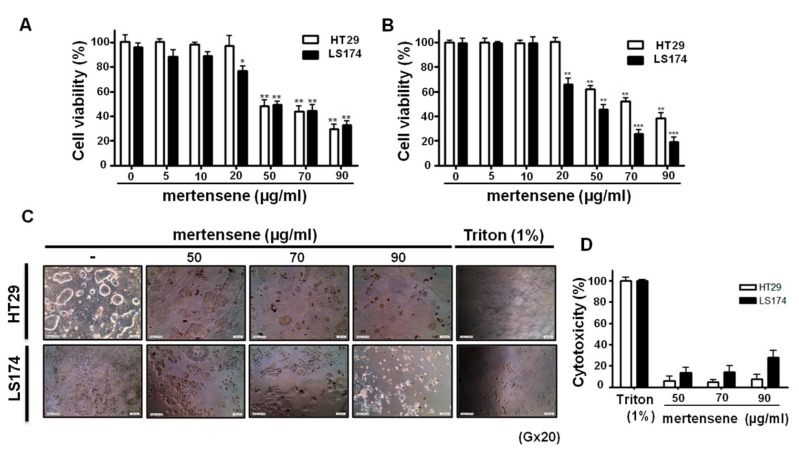
Mertensene inhibits HT29 and LS174 cell viability. Cells were treated with increasing concentrations of mertensene (50, 70, 90 µg/mL) for 72 h. Cell viability was analyzed by MTT assay (**A**) and trypan blue method (**B**). The morphological changes were detected by microscopic observation (**C**) and the cytotoxicity was evaluated by LDH assay (**D**). Values are means ± S.D. from three independent experiments. Statistical differences were analyzed with Student’s *t*-test (* *p* < 0.05, ** *p* < 0.01, *** *p* < 0.001).

As the state of the tumor suppressor *p53* is pivotal for the response of tumor cells to anticancer therapy [27], we investigated whether mertensene could affect the viability of human colon adenocarcinoma LS174 (wild type *p53*) and HT29 which is a *p53* mutant cell line. We examined the effects of increasing concentrations of mertensene (0–90 µg/mL) on the viability of HT29 and LS174 cells for 72 h using two complementary methods, the MTT assay and trypan blue dye to exclude any artifacts that may come from interaction of mertensene with MTT, which could be directly reduced by this compound. Interestingly, we found that both methods showed similar results and that mertensene significantly reduced the viability of LS174 and HT29 cells in a dose-dependent manner, independently of their *p53* status. The efficient doses were between 50 and 90 µg/mL and the IC_50_ values of mertensene were 56.50 ± 8.68 µg/mL for HT29 cells and 49.77 ± 4.51 µg/mL for LS174 cells (Figure 2A,B).

### 2.3. Mertensene Did Not Induce Plasmatic Membrane Damage

In order to verify if cell death induced by mertensene is due to damage of plasmatic membrane, we measured the Lactate Dehydrogenase (LDH) activity in culture supernatant of mock and treated HT29 and LS174 cells with the effective doses of mertensene (50, 70 and 90 µg/ mL) for 72 h using the LDH assay. Figure 2D shows that compared to the positive control (100% toxicity, Triton 1%), the LDH leakage which is proportional to the number of lysed cells is more important in mertensene-treated LS174 than in HT29 cells. The percentages of cell cytotoxicity ranged from 5.7 ± 2.4% to 7.8 ± 2.8% in HT29 cells and from 13.9 ± 6.2% to 29.3 ± 3.3% in LS174 cells. Thus, mertensene is ~2.4 to ~3.7 fold more cytotoxic against LS174 than HT29 cells. Based on these results and since mertensene is able to inhibit the viability of HT29 cells that express a *p53* mutated form (R273H) and characterized by their increased proliferation, metastatic potential and reduced apoptosis, we have chosen this cell line for further investigations.

### 2.4. Mertensene-Induced G2/M Cell Cycle Arrest Is Mediated by Related Regulatory Effectors

**Figure 3:**
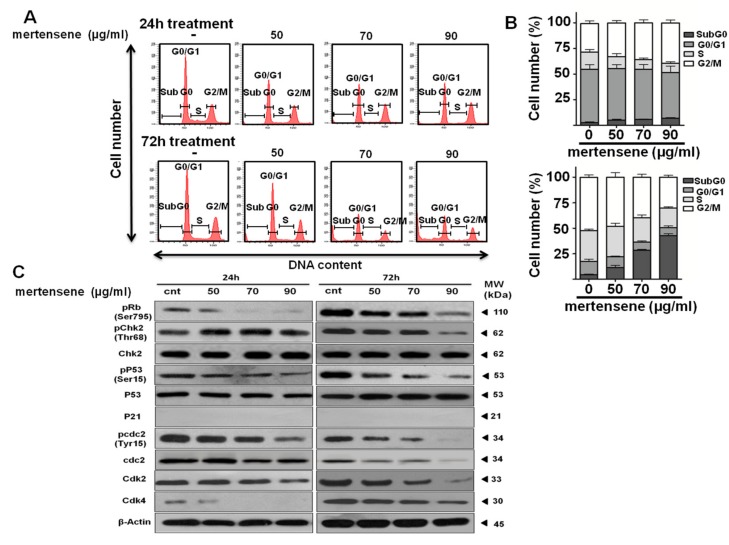
Mertensene induces G2/M cell cycle arrest in HT29 cells through various cell cycle regulatory proteins. Cells were mock-treated with vehicle or mertensene (50, 70, 90 µg/mL) for 24 h and 72 h. (**A**) Cell cycle distribution was assessed by flow cytometry after propidium iodide staining; (**B**) The percentages of cells in the different phases of the cell cycle were represented; (**C**) Whole cell lysates were analyzed on SDS-PAGE gel and probed with the indicated antibodies for Western blot analysis. β-Actin is shown as protein loading control. The data are representative of three independent experiments.

Suppression of tumor cell growth can be caused either by arrest of cell cycle progression or induction of apoptosis or both [28]. In order to examine mertensene’s effect on cell cycle progression and related effectors, HT29 cells were treated with 50, 70 and 90 µg/ mL of the compound for 24 h and 72 h. Cell cycle distribution was analyzed by flow cytometry. Figure 3A,B showed that after 24 h of treatment, mertensene (at 50, 70 and 90 µg/mL) induced accumulation of HT29 cells in G2/M phase (from 27.8% (control) to 33%, 35.9% and 39.4%, respectively) with a concomitant decrease in the number of cells in G0/G1 (from 52% (control) to 50.2%, 48.8% and 44.6%, respectively) and S phase (from 16.8% (control) to 11.6%, 9.5% and 9%, respectively). Prolonged exposure of cells (72 h) to increasing concentrations of mertensene induced disturbance of cell cycle phases and correlated with an increase, in a dose-dependent manner, in the number of cells in sub-G0 phase from 2.59, ~6.48, ~ to 9.73 fold which is indicative of apoptotic population (Figure 3A,B).

To explore the mechanism by which mertensene induced G2/M cell cycle arrest, we examined the expression level of cell-cycle key regulatory proteins by Western blotting. Figure 3C showed a dose-dependent decrease in the expression level of cdk2, cdk4 proteins and in the phosphorylated forms of retinoblastoma protein RB (pRb), pcdc2 and pchkp2 after 72 h of mertensene treatment. Moreover, mertensene decreased the phosphorylation of *p53* at Ser15, while it did not affect the expression of *p53*. The expression of p21, a target of *p53* and a cyclin-dependent kinase inhibitor could not be detected in these cells.

Thus, mertensene arrested HT29 cells in the G2/M phase through negative modulation of different cell cycle regulating proteins.

### 2.5. Mertensene Induces Apoptotic Cell Death in HT29 Cells

**Figure 4:**
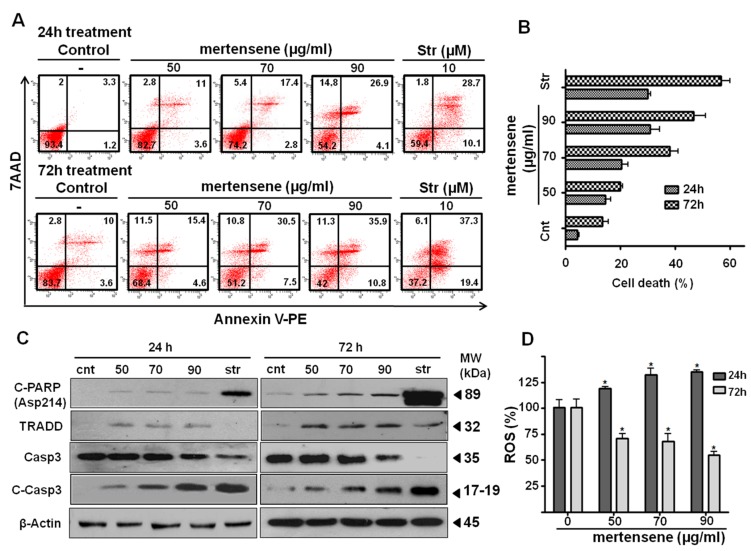
Mertensene induces apoptotic cell death and modulates reactive oxygen species (ROS) accumulation in HT29 cells. Cells were mock- or mertensene-treated at concentrations of 50, 70, 90 µg/mL for 24 h and 72 h. (**A**) Flow cytometric analysis of apoptotic cells after annexin V/7-AAD staining of mock- and mertensene-treated cells. Staurosporine was used as positive control of apoptosis. Bottom left quadrant represents the live cells that are Annexin V−/7AAD−, early apoptotic cells (Annexin V+/7AAD−; bottom right quadrant), late apoptotic or necrotic cells (Annexin V+/7AAD+; upper right quadrant), and dead cells (Annexin V−/7AAD+; upper left quadrant). (**B**) Data show a dose-dependent increase in the number of apoptotic cells in HT29 cells after treatment with mertensene. (**C**) Protein extracts from whole cell lysates were analyzed by Western blot using the indicated antibodies. Anti-actin was used as loading control. (**D**) ROS production was evaluated by detection of fluorescence using the fluorogenic probe CMH2DCFDA after 24 and 72 h of treatment with increasing concentrations of mertensene. The results were normalized to mock-treated cells (control). Data represent the means ± SD of three separate experiments. Statistical differences were analyzed with Student’s *t*-test (* *p* < 0.05).

The morphological changes observed after mertensene treatment (Figure 2C) and the increase in subG0 population suggested that mertensene induces apoptotic cell death. We quantified the percentage of apoptotic cells by flow cytometry after Annexin-V staining. Interestingly, treatment with increasing concentrations of mertensene induced a dose- and time-dependent increase of Annexin V-positive staining cells (Figure 4A,B). Compared to mock-treated cells, mertensene treatment at concentrations of 50, 70 and 90 µg/mL resulted in 14.6%, 20.2% and 31% apoptotic cell population, respectively after 24 h. The percentage of apoptotic cells increased from 13.6% (mock) to 20%, 38% and 46.7%, respectively, in mertensene-treated cells for 72 h.

We then assessed whether mertensene-induced apoptotic cell death in HT29 cells could be associated to caspases activation. As shown in Figure 4C, Western blot analysis revealed that treatment of cells with mertensene induced a dose- and time-dependent activation of caspase-3 and PARP cleavage. This compound increased the expression level of the death receptor-associated protein TRADD. Taken together, these results suggested that mertensene triggered apoptosis in HT29 cells through intrinsic and extrinsic pathways.

### 2.6. Mertensene Modulates ROS Production

It is well documented that the increase of reactive oxygen species (ROS) could cause cell apoptosis [29]. We thus assessed the effect of mertensene on the intracellular redox status of HT29 cells after 24 and 72 h treatment. Interestingly, we found that although mertensene induced ROS accumulation after 24 h of treatment, it inhibited the intracellular amount of ROS in HT29 cells after 72 h. This result suggests that mertensene modulates ROS as a treatment exerting pro-oxidant function that sensitizes HT29 cells to early event of apoptosis. In contrast, at late phase, when the intracellular levels of ROS are important, mertensene could act as an antioxidant to deplete ROS from HT29 cells (Figure 4D).

### 2.7. ERK-1/-2, AKT and NF-κB Activation Contributes to Mertensene-Induced Inhibition of HT29 Cell Viability

Subsequently, we explored the signaling cascades that may contribute to inhibitory effect of mertensene in HT29 cells. We tested the activation of pro survival ERK-1/-2, AKT protein kinases and NF-κB that play a critical role in cell survival [30,31].

**Figure 5:**
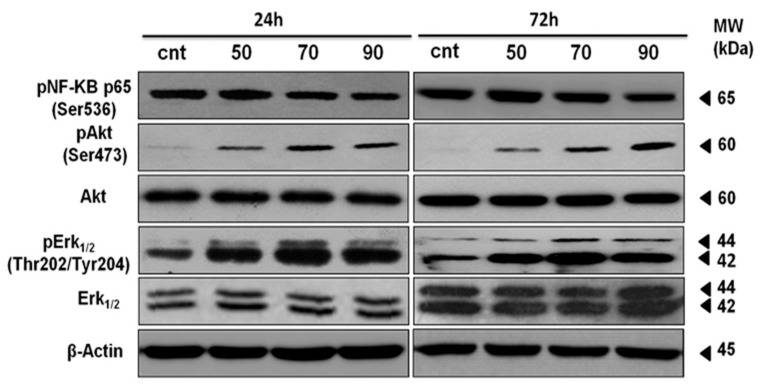
Mertensene induces ERK-1/-2, AKT and NF-κB activation in HT29 cells. Cells were treated with vehicle or with mertensene (50, 70 and 90 µg/mL) for 24 h and 72 h. Protein extracts (30 µg) prepared were analyzed by Western blotting using the indicated antibodies. β-Actin was used as a loading control.

As shown in Figure 5, mertensene caused an increase in ERK-1/-2 and AKT phosphorylation associated to a slight decrease in phosphorylated form of NF-κB in a dose and time-dependent manner. This result suggests that the anti-proliferative activity of mertensene is mediated, at least in part, through modulation of ERK-1/-2, AKT and NF-κB pathways recognized as major regulators of cell survival.

## 3. Discussion

Colorectal cancer is one of the leading causes of cancer mortality worldwide [2]. Despite the development of several therapies for its treatment, there is still no effective cure for patients with advanced stages of the disease and the prognosis for advanced tumors remains reserved. Therefore, more studies are still needed to develop non-toxic new anti-tumor approaches with minimal side effects and targeting major events leading to carcinogenesis. The marine environment constitutes a prolific source of therapeutic compounds that exhibit anti-tumor activities. Terpenoids are good example of marine natural products that provided a vast array of molecular architectures with interesting potential in pharmaceutical and biotechnological applications. This class of compounds has demonstrated effectiveness against tumor cells and has been found to be useful in the prevention and therapy of several human cancers [10,32,33,34]. Cheng and collaborators reported the identification of two novel dinormonoterpenes Mollisolactones A and B from the soft coral *Sinularia mollis* that exhibited anti-proliferative activities against different cancer cell lines [35].

The anti-cancer activity of nine halogenated monoterpenes isolated from the red alga *P. cartilagineum* has been highlighted on murine colon adenocarcinoma CT26 cells, human colon adenocarcinoma SW480 cells, human cervical adenocarcinoma HeLa cells and human malignant melanoma SkMel28 cells [13]. More recently, Sabry et al. [36] reported the identification of four halogenated monoterpenes purified also from the red alga *P. cartilagineum* as a cytotoxic agents in human leukemia, lung, colon cancer cells and mouse neuro-2a cell lines [36]. However, the underlying anti-tumor mechanisms of these reported halogenated monoterpenses are still uncovered.

In this study, we extracted and characterized the halogenated cyclic monoterpene mertensene from the red alga *P. capillacea*. We demonstrated that this compound inhibits the viability of human colorectal adenocarcinoma cells by inducing cell cycle arrest and apoptosis through regulating key effectors and survival pathways.

Initially, we observed that mertensene induced a dose-dependent inhibition effect on the viability of human colon adenocarcinoma LS174 cells (*p53* wild type). It is well documented that the tumor suppressor *p53* is an attractive target in cancer therapy [37]. Most of the tumor malignancies are associated with mutation of *p53* gene and the state of *p53* is pivotal for the response of tumor cells to anticancer therapies [27]. Interestingly, mertensene was able to affect the viability of HT29 (*p53* mutant) colon adenocarcinoma cells that express a *p53* mutated form (R273H), a very well-known hot spot mutation in tumors. This result suggests that mertensene effects on the viability of colon cancer cells did not rely on *p53*. In agreement with our data, other studies demonstrated a *p53*-independent apoptosis in colon cancers [38,39]. Thapa et al. [40] reported that LYR-8, a hexahydrocannabinol analog, led to apoptosis in colon cancer cells HCT116 (wild-type) and HT29 (*p53* mutant). This effect occurred irrespective of *p53* status [40].

Since R273H mutant cells have been characterized for their increased proliferation, their metastatic potential and their reduced apoptosis, we characterized the mechanisms associated with the anti-tumor activity of mertensene in HT29 (*p53* mutant) colon cancer cells.

The anti-tumor effect of many drugs has been associated with a cell cycle disturbance and cell apoptosis. Cell cycle governs the transition from quiescence (G0) to cell proliferation, and through its checkpoints, ensures the fidelity of the genetic transcript [41]. In colorectal cancer, cell cycle deregulation is common and contributes to tumorigenesis. In our study, we demonstrated that mertensene suppressed cell cycle progression, at G2/M phase through a negative modulation of phosphorylated forms of *p53*, retinoblastoma protein (Rb), cdc2 and chkp2 and reduced expression level of cyclin-dependent kinases CDK2 and CDK4.

It is well documented that deregulated apoptosis often leads to tumor formation and cell drug resistance [42]. Therefore, using compounds that induce apoptosis is a promising approach in cancer therapy [43,44,45]. Apoptosis can be achieved through the family of cysteine proteases, the caspases [28,46,47]. Caspases are classically activated by intrinsic (mitochondrial-mediated) and extrinsic (death receptors-mediated) pathways involving cytochrome C and cell surface death receptors, leading to caspase-3 activation, which cleaves poly (ADP-ribosyl) polymerase (PARP) leading to cell death [48,49].

Interestingly, by flow cytometry using annexin V binding assay, we demonstrated that mertensene triggers a caspase dependent apoptosis in HT29 cancer cells. This effect implies increased expression level of death receptor-associated protein TRADD, activation of caspase-3 and the cleavage of PARP, a hallmark of apoptosis. Our results are in accordance with several studies reporting the caspase-3-mediated apoptosis and PARP cleavage in HT29 cells as a mechanism responsible for antitumor effects induced by several natural compounds in these cells [50,51]. In agreement with our data, Meng et al. [39] described that trichostatin A induces G2/M cycle arrest and apoptosis in both colorectal cancer cells HCT116 and HT29 via mitochondrial pathway by causing dissipation of mitochondrial membrane, caspase-3 activation and PARP cleavage [39]. More recently, Dyshlovoy et al. [52] reported that the marine triterpene glycoside frondoside A caused cell cycle arrest and induced prostate cancer cell apoptosis through cleavage of PARP and caspase-3 [52].

Given the critical role of ERK-1/-2, AKT and NF-κB signaling pathways in controlling cellular growth and survival in colorectal cancer [53,54,55], we investigated whether mertensene-induced inhibition of HT29 cell viability could modulate these proteins. Interestingly, mertensene-induced HT29 cell death was associated with the activation of ERK-1/-2, AKT and a moderate inhibition of NF-κB signaling pathways. ERK-1/-2, AKT and NF-κB signaling pathways are major regulators of cell survival, proliferation, metabolism, and motility that are commonly activated in colorectal cancer. Constitutive NF-κB activation plays a key role in the development and progression of colorectal cancer [56]. Several anticancer therapeutic drugs prevented colorectal cancer progression through inhibition of the NF-κB signaling [57].

It is well documented that in contrast to their advantageous role in both cell proliferation and survival, sustained activation of ERK-1/-2 and AKT is implicated in the induction of cell death [58]. Indeed, constitutive activation of ERK-1/-2 pathway initiates apoptosis and cell cycle arrest [59]. Our results are in accordance with those reporting that Asperolide A and Wentilactone A extracted from the marine-derived endophytic fungus *Aspergillus wentii* EN48 inhibited lung carcinoma cell proliferation by inducing G2/M cell cycle arrest through the activation of ERK signaling [60,61]. Different anticancer agents triggered cell apoptosis through the modulation of intracellular ROS levels [62] that could enhance the activation of the ERK-1/-2 and AKT pathways [63].

In HT29 cells, even if mertensene induces AKT and ERK-1/-2 activation that persists after 72 h, the ROS formation outcome was different depending on the time duration of the treatment. This suggests that mertensene could act as a pro-oxidant to induce cell death and/or as an antioxidant to deplete ROS from HT29 cells. Our data are also consistent with those reported by Nogueira et al. [64], demonstrating that Akt activation increases intracellular ROS and sensitizes cells to ROS-mediated apoptosis by mediating oxygen consumption [64].

Thus, the use of combined targeted strategy based on the interference with multiple cross-talking pathways that are involved in cell growth and survival process, such as PI3K/AKT, ERK-1/-2 and NF-κB, constitutes an attractive concept and holds much promise for anticancer therapy.

## 4. Experimental Section

### 4.1. Extraction and Isolation of Mertensene

Specimens of *Pterocladia capillacea* were collected from the Atlantic coasts of Morocco in the region of “Al Jadida” in 2009 and were kindly identified by Professor Saloua SADOK at the Laboratory of Biodiversity and Biotechnology, Institut National des Sciences et Technologies de la Mer, Salammbô, Tunisia. Voucher specimens (PC01) have been deposited at the Unité de Toxines Alimentaires, Institut Pasteur de Tunis. The algal specimens were washed with fresh water, dried at room temperature and powdered (1.97 kg dry wt). The powdered algal biomass was extracted with mixtures of CH_2_Cl_2_/MeOH (3:1) and subsequently the solvents were evaporated under vacuum to afford a dark green oily residue (8.8 g). The organic extract was subjected to vacuum column chromatography on silica gel, using cyclohexane with increasing amounts (10%) of EtOAc, followed by EtOAc with increasing amounts (10%) of MeOH as mobile phase, to obtain 11 Fractions (1–11). Fraction 2 (20% EtOAc in cyclohexane) was further purified by gravity column chromatography on silica gel using mixtures of cyclohexane/EtOAc of increasing polarity to yield 9 Fractions (2a–2i), among which 2b (1% EtOAc in cyclohexane, 100.2 mg, 0.005% dry wt) was identified as mertensene in pure form, as determined by HPLC analysis.

### 4.2. Chemical Studies

NMR spectra were recorded on Bruker AC 200 and Bruker DRX 400 spectrometers. Chemical shifts are given on a *δ* (ppm) scale using TMS as internal standard. The 2D experiments (HSQC, HMBC, COSY, NOESY) were performed using standard Bruker pulse sequences. Low resolution EI mass spectra were measured on a Hewlett Packard 5973 mass spectrometer. Column chromatography separations were performed with Kieselgel 60 (Merck, Kenilworth, NJ, USA). TLC were performed with Kieselgel 60 F_254_ (Merck aluminum support plates) and spots were detected after spraying with 15% H_2_SO_4_ in MeOH reagent and heating at 100 °C for 1 min.

### 4.3. Pharmacological Studies

#### 4.3.1. Cell Cultures

Human colon adenocarcinoma LS174 (CL-188, ATCC, Manassas, VA, USA) and HT-29 (HTB-38, ATCC, Manassas, VA, USA) cell lines were cultured in Dulbecco’s modified Eagle’s medium (DMEM) supplemented with 10% heat-inactivated fetal bovine serum (FBS; GIBCO) and antibiotics (100 μg/mL streptomycin and 100 U/mL penicillin). Cultures were maintained at 37 °C in humidified 5% CO_2_ atmosphere.

#### 4.3.2. Cell Viability Assays

Cell viability was assessed by 3-(4,5-dimethylthiazol-2yl-)-2,5-diphenyl tetrazolium bromide (MTT, Sigma) and trypan blue exclusion tests.

MTT assay: Cell viability was measured by MTT reduction via mitochondrial oxidation of living cells. Cells were seeded into 96-well-plate at 10^4^ cells/mL, incubated for 24 h, and then treated with various concentrations of mertensene (50 µg/mL (156 µM), 70 µg/mL (218 µM) and 90 µg/mL (230 µM)) and further incubated for 72 h. Cells were then treated with MTT reagent (1 mg/mL) and incubated for 3 h at 37 °C and 5% CO_2_. Dimethyl sulfoxide (DMSO, Sigma-Aldrich, St. Louis, MO, USA) was added to dissolve the blue formazan and the absorption was measured at 540 nm using a microplate reader (MULTISKAN, Labsystems, Waltham, MA, USA). Results were expressed as percentage of the viable cell number in treated cells relative to mock-treated cells.

Trypan blue test: The mock and treated cells were stained with 0.4% (*w/v*) trypan blue solution and counted under an optical microscope. Cells failing to exclude the dye were defined as dead cells. The cell viability (%) was calculated using the following formula: (total viable cells (unstained) of treated group/total viable cells of the negative control group) × 100.

#### 4.3.3. LDH Cytotoxicity Assay

Cellular toxicity was assessed through lactate dehydrogenase (LDH) leakage into the culture medium using the LDH Cytotoxicity Detection Kit-PLUS test (Roche Applied Science, Mannheim, Germany) according to the manufacturer’s protocol. Cells were seeded at 10^5^ cells/mL and cultured with different concentrations of mertensene (50 µg/mL (156 µM), 70 µg/mL (218 µM) and 90 µg/mL (230 µM)) or Triton X-100 which was used as positive control for 100% of cytotoxicity. The plates were incubated for 24 h and 72 h. LDH activity was evaluated by measuring the absorbance at 490 nm. The percentage of cytotoxicity was calculated relatively to the positive (1% Triton X-100 treatment) and negative controls (the spontaneous LDH release in the mock-treated cells) as follows: Cytotoxicity (%) = (experimental value − negative control)/(positive control − negative control) × 100.

#### 4.3.4. Cell Cycle Analysis

Cells were treated with various concentrations of mertensene for 24 h and 72 h. The cells were harvested at 1000 rpm, washed twice with 1× ice-cold PBS containing 2% bovine albumin serum (BSA) (Sigma), resuspended in hypertonic solution (20 mM HEPES, pH = 7.2; 0.16 M NaCl; 1 mM EGTA; 0.05% Triton X-100) and placed immediately on ice. Cells were pelleted and resuspended in 500 µL of propidium iodide (PI)/RNase staining solution (Cell Signaling Technology; Danvers, MA, USA). After incubation for 30 min in the dark at 37 °C, cells were observed on a Becton Dickinson FACScanto II flow cytometer (BD Biosciences, San Jose, CA, USA) and further analyzed with BD FACSDiva 6 software (BD Biosciences, San Jose, CA, USA). The PI fluorescence signal at FL2-A peak versus counts was used to determine cell cycle distribution.

#### 4.3.5. Cell Apoptosis Analysis

The rate of apoptotic cells was assessed by flow cytometry using the annexin V/7-amino-actinomycin D (7-AAD) apoptosis detection kit (BD Biosciences, San Jose, CA, USA) according to the manufacturer’s protocol. Briefly, cells were treated with various concentrations of mertensene (50 µg/mL (156 µM), 70 µg/mL (218 µM) and 90 µg/mL (230 µM)) for 24 h and 72 h. Staurosporine (10 µM) was used as a positive control. Cells were collected by centrifugation and resuspended in 100 μL of binding buffer (1×). After staining with 4 µL of annexin V conjugated to phycoerythrin (PE) and equal volume of 7-AAD, the samples were incubated at room temperature in the dark for 15 min. Cell death was assessed on a Becton Dickinson FACScanto II flow cytometer and analyzed with BD FACSDiva 6 software (Becton Dickinson, San Jose, CA, USA). Apoptosis was quantitatively evaluated by measuring the proportion of annexin V-positive cells regardless of their staining with 7-AAD.

#### 4.3.6. Detection of Intracellulaar Reactive Oxygen Species (ROS)

The intracellular production of ROS was quantified using a cell-permeable fluorogenic probe, CMH2DCF-DA (Life Technologies, Camarillo, CA, USA). This dye freely permeates the plasma membrane where it is deacetylated by cellular esterases to a non-fluorescent compound, which is later oxidized by ROS into 2′,7′-dichlorofluorescein (DCF).

HT29 cells were seeded in 96-well plates (2000 cells/well) and treated with 50, 70 and 90 µg/ mL of mertensene for 24 h and 72 h. Cells were washed with PBS (1×), resuspended in HBSS (GIBCO BRL, Karlsrahe, Germany) and incubated with CMH2DCFDA (10 µM) at 37 °C for 30 min in dark. Fluorescence was detected with excitation and emission wavelength at 492 nm and 517 nm respectively.

#### 4.3.7. Western Blot Analysis

After treatment with mertensene, HT29 cells were washed twice with PBS and subsequently lysed with 100 µL 1× Laemeli buffer at room temperature. The whole protein content of cell lysates was determined by the BCA protein assay (Bicinchoninic Acid Protein Assay kit, Sigma). Equal amounts of the total protein samples were separated by 10% sodium dodecyl sulphate (SDS) polyacrylamide gel electrophoresis, transferred onto PVDF membranes (Immobilon-Millipore), probed with primary antibodies and incubated with a horseradish peroxidase-conjugated anti-IgG in a blocking buffer for 1 h or 2 h. Visualization of protein expression was performed using enhanced chemiluminescence (ECL) (GE Healthcare, Amersham, UK) and exposure to X-ray film (Sigma Aldrich). The polyclonal antibodies specific to TRADD, Cleaved PARP, Caspase-3, Cleaved Caspase-3, Cdk2, Phospho-cdc2 (Tyr15), Phospho-Rb (Ser795), Chk2, Phospho-Chk2 (Thr68), Phospho-NF-κB p65 (Ser536), total Akt, Phospho-p44/42 MAPK (ERK-1/-2) (Thr202/Tyr204), and the monoclonal antibodies specific to cdc2, Phospho-Akt (Ser473), total p44/42 MAPK (ERK-1/-2), β-Actin and Cdk4 were purchased from and Cell Signaling Technology (Danvers, MA, USA).

### 4.4. Statistical Analysis

Graph-pad prism was used to evaluate the results as the mean ± SD and statistical analyses were made by Student’s *t*-test. *p*-values < 0.05 were considered to be statistically significant.

## 5. Conclusions

In summary, the present study demonstrated that mertensene exhibits an antiproliferative effect on two human colorectal adenocarcinoma cell lines (HT29 and LS174), independently of their *p53* status. Moreover, treatment of HT29 cells with mertensene significantly inhibits cell viability and induces cell apoptosis via modulation of several cellular effectors and major survival pathways, ERK-1/-2, AKT and NF-κB pathways. Actually, several compounds targeting these pathways are validated or under clinical phases development. Mertensene could become one of these promising new anticancer drugs that are specific for their targets, less toxic and well tolerated than the conventional cytotoxic chemotherapeutic agents used in the treatment of colorectal cancer [53].
